# Coiled-Coil Domain-Containing 68 Downregulation Promotes Colorectal Cancer Cell Growth by Inhibiting ITCH-Mediated CDK4 Degradation

**DOI:** 10.3389/fonc.2021.668743

**Published:** 2021-04-22

**Authors:** Cong Wang, Hongyan Li, Lei Wu, Xueli Jiao, Zihui Jin, Yujie Zhu, Ziling Fang, Xiaodong Zhang, Haishan Huang, Lingling Zhao

**Affiliations:** ^1^ Key Laboratory of Laboratory Medicine, Ministry of Education, School of Laboratory Medicine and Life Sciences, Wenzhou Medical University, Wenzhou, China; ^2^ Department of General Surgery, Heze Municipal Hospital, Heze, China; ^3^ Department of Colorectal anal surgery, The First Affiliated Hospital of Wenzhou Medical University, Wenzhou, China

**Keywords:** CCDC68, colorectal cancer, CDK4 degradation, ITCH, cell proliferation

## Abstract

Coiled-coil domain-containing 68 (CCDC68) plays different roles in cancer and is predicted as a tumor suppressor in human colorectal cancer (CRC). However, the specific role of CCDC68 in CRC and the underlying mechanisms remain unknown. Here, we showed that CCDC68 expression was lower in CRC than that in corresponding normal tissues, and CCDC68 level was positively correlated with disease-free survival. Ectopic expression of CCDC68 decreased CRC cell proliferation *in vitro* and suppressed the growth of CRC xenograft tumors *in vivo*. CCDC68 caused G0/G1 cell cycle arrest, downregulated CDK4, and upregulated ITCH, the E3 ubiquitin ligase responsible for CDK4 protein degradation. This increased CDK4 degradation, which decreased CDK4 protein levels and inhibited CRC tumor growth. Collectively, the present results identify a novel CDK4 regulatory axis consisting of CCDC68 and ITCH, which suggest that CCDC68 is a promising target for the treatment of CRC.

## Introduction

Colorectal cancer (CRC), one of the three most common cancers worldwide, has the third highest incidence rate (10%) and the second highest mortality rate (9.4%), and is therefore a considerable threat to human life and health (1). Currently, approximately one in ten CRC patients die from the disease each year despite undergoing treatment (1). Understanding the molecular mechanisms underlying the occurrence and development of CRC and exploring new approaches to the treatment of CRC are important research objectives.

The occurrence and development of CRC are mediated by a complex process that involves multiple pathways (2, 3), such as the EGFR, Wnt/β-catenin, TGF-β, and Sonic Hedgehog pathways. The factors and/or genes involved in these pathways may be potential therapeutic targets in CRC (4, 5). The combination of panitumumab, a human monoclonal antibody against EGFR, with supportive therapy is a common strategy for the treatment of metastatic CRC (6). Several inhibitors of the Wnt/β-catenin signaling pathway have been developed for the treatment of CRC (7). Identifying and understanding the factors and/or genes implicated in CRC occurrence and development would facilitate the early diagnosis or treatment of CRC patients.

Coiled-coil domain-containing (CCDC) proteins are a large family of proteins possessing a unique α-helical coiled coil that mediates multiple functions, including cytoskeleton formation, regulation of cell polarity and movement, transportation of intracellular substances, molecular recognition, and signal transduction (8). Many members of this family are involved in the progression of multiple cancers, such as CCDC43 in gastric cancer (9), CCDC8 in lung cancer (10), and CCDC34 in bladder cancer (11). CCDC68, a member of the CCDC family, is an important component of mother centriole subdistal appendages; it is a centrosome protein and is thus involved in cell cycle progression (12–14). CCDC68 also plays a role in cancer. Radulovich et al. found that CCDC68 is downregulated and acts as a tumor suppressor in pancreatic ductal adenocarcinoma (15). However, CCDC68 is upregulated in non-small cell lung cancer (NSCLC) and promotes cell proliferation (16). Therefore, the role of CCDC68 in cancer remains controversial. There is only one article reporting that CCDC68 is downregulated in 89% of patients with primary CRC, suggesting that CCDC68 might be a new candidate tumor suppressor gene in CRC (17). The specific biological role of CCDC68 in CRC and the underlying mechanism remain largely unclear.

In this study, we demonstrated that CCDC68 suppresses CRC cell proliferation *in vitro* and *in vivo* by promoting ITCH transcription. This was mediated by upregulation of the transcription factor RXRα and alterations of cyclin-dependent kinase (CDK)-4 protein degradation. We showed that CCDC68 downregulation promoted the growth of CRC cells through the RXRα/ITCH/CDK4 axis, thereby identifying a new mechanism underlying the development of CRC and providing a theoretical basis for targeted therapy for CRC.

## Materials and Methods

### Plasmids, Antibodies, and Reagents

The HA-CCDC68 plasmid, a set of shRNA plasmids specifically targeting ITCH, and the Flag-RXRα plasmid were purchased from MiaoLingBio (Wuhan, China). The pEGFP-CDK4 plasmid was constructed as described in our previous study (18). The luciferase reporter driven by the ITCH promoter was constructed by inserting the ITCH promotor sequence into the pGL3-basic vector (E1751; Promega) with the primers (F) 5′;-TCTATCGATAGGTACCTGACTTTCCAGATGGCAAAATACT-3′; and (R) 5′;-CCGGAATGCCAAGCTTTTCGCCCACGGGGGTTTA-3′;. Antibodies against CDK2 (sc-6248), CDK4 (sc-260), CDK6 (sc-177), cyclinD1 (sc-20044), P21 (sc-397), and P27 (sc-1641) were purchased from Santa Cruz Biotechnology (Santa Cruz, CA, USA). Antibodies against CDC37 (4793S), cyclin E2 (4132S), HA (3724S), BTRC (11984S), and RXRα (3085S) were purchased from Cell Signaling Technology (Boston, MA, USA). Antibodies against α-Tubulin (ab7291), FBXO4 (ab230302), and SOCS7 (ab224589) were purchased from Abcam (Cambridge, UK). Antibodies against CCDC68 (PA5-61687) and FZR1 (34-2000) were purchased from Invitrogen (Grand Island, NY, USA). Antibody against ITCH (20920-1-AP) was purchased from Proteintech (Chicago, IL, USA). The chemicals cycloheximide (CHX) and MG132 were purchased from Calbiochem (San Diego, CA, USA).

### Clinical Specimens

This study was approved by the Ethics Committee of Wenzhou Medical University. Tumor tissues and corresponding adjacent normal tissues were obtained from patients with colorectal cancer treated at the First Affiliated Hospital of Wenzhou Medical University (Zhejiang, China). A total of 150 pairs of tumor and normal tissues were collected and confirmed by histological and pathological diagnosis (**Table S1**). Each specimen was divided into two parts: RNA was extracted from one third of the material, and cDNA was synthesized and stored at -80°C until analysis. Two thirds of the material were fixed in formalin, embedded in paraffin, and stored at room temperature.

### Cell Culture and Transfections

The human CRC cell lines HCT116 and HT29 were obtained from the Cell Bank of Shanghai Institute of Biochemistry and Cell Biology, Chinese Academy of Sciences. SW480 cells were bought from ATCC. HCoEpiC and RKO cells were bought from Cobioer (Nanjing, China). HCT116 and HT29 cells were maintained in McCoy’s 5A medium supplemented with 10% fetal bovine serum (FBS); RKO and SW480 cells were cultured in 1640 medium (Gibco, 11875–093) containing 10% FBS; and HCoEpiC cells were cultured in minimum essential medium (MEM; Gibco, 11095–080) containing 10% FBS. All cells were grown in a 5% CO_2_ cell culture incubator at 37°C. For stable cell line construction, HCT116 and RKO cells were transfected with plasmids using PolyJet™ DNA In Vitro Transfection Reagent (SignaGen Laboratories, SL100688). After 48 h, cells were subjected to selection with puromycin (4–6 μg/mL; J593, Amresco Inc) or G418 (1000–1500 μg/mL; sc-29065, Dallas, TX, USA) according to the antibiotic resistance of different transfected plasmids.

### Lentivirus Packaging and Infection

Lentivirus packaging and infection experiments were performed as described previously (18). HA-CCDC68 stable expression cells and vector control cells were constructed by lentivirus infection. Briefly, two packaging vectors, 1.2 μg pMD2.G (12259, Addgene) and 1.2 μg psPAX2 (12260, Addgene), and 2.0 μg HA-CCDC68-PLVX-puro or HA- PLVX-puro plasmid, were transfected into 293T cells. The viral supernatants were then collected after 48 h, filtered, and used to infect HCT116 and RKO cells. Stable cell lines were screened by puromycin.

### Immunohistochemistry (IHC)

IHC assays were performed to detect CCDC68 expression in formalin fixed paraffin embedded CRC specimens obtained from humans. An antibody specific against CCDC68 (PA5-61687; Invitrogen, Grand Island, NY, USA) was used for IHC staining, which was performed using a kit from Boster Bio-Engineering Company (SA1022; Wuhan, China). Immunostained images were captured with the Nikon Eclipse Ni microsystem (DS-Ri2) and analyzed with Image-Pro Plus version 6.0 (Media Cybernetics, Rockville, MD, USA) by calculating the integrated optical density (IOD) of each stained area (IOD/area). At least five images per specimen were counted.

### Western Blotting

The cells were lysed with a lysis buffer containing 10 mM Tris-HCl, 1 mM Na_3_VO_4_, and 1% SDS (pH 7.4) on ice. The samples were then collected and heated at 100°C for 5 min, and nucleic acids were broken with ultrasound. Protein samples were separated on SDS-PAGE gels, followed by transfer to polyvinylidene fluoride membranes (Bio-Rad, Hercules, CA, USA). The membranes were blocked with 5% non-fat milk and probed with the indicated primary antibodies overnight at 4°C, and then incubated with AP-conjugated secondary antibody. The ECF western blotting system (RPN5787; GE Healthcare, PA, USA) was used to detect the protein signal, and images were captured using a phosphorimager (Typhoon FLA 7000, GE Healthcare) (19).

### Anchorage-Independent Growth Assay

The potential inhibitory effect of CCDC68 on the anchorage-independent growth of human CRC cells was assessed in the HCT116 and RKO cell lines. First, 0.5% agar in Basal Medium Eagle (BME) containing 10% FBS was used to cover the bottom layer of 6-well plates. Then, 1 × 10^4^ HCT116 (Vector), HCT116 (CCDC68), RKO (Vector), and RKO (CCDC68) stable transfectants were suspended in 1 mL of 0.33% agar in 10% FBS-BME and seeded on the bottom layer. After culturing in a 5% CO_2_ incubator for 2–4 weeks, the number and size of cell colonies were determined under a microscope (DMi1; Leica Microsystems, Germany) (20).

### Cell Proliferation

A Cell Titer-Glo Luminescent Cell Viability Assay kit (G7572; Promega) was used to determine the effect of CCDC68 on the proliferation of CRC cells. The assay was performed as described previously (18). Briefly, 2000 HCT116 (Vector), HCT116 (CCDC68), RKO (Vector), and RKO (CCDC68) cells were seeded into each well of 96-well plates. After adherence, the cells were synchronized for 12 h by exposure to 0.1% FBS medium and then cultured in complete medium for the indicated days. Cell viability was detected at 1, 3, and 5 days using a Centro LB 960 luminometer (Berthold Technologies, Berthold, Germany). Cell proliferation rate was defined as the relative absorbance of cells cultured for 3 and 5 days versus that of cells cultured for 1 day. Each experiment was repeated at least three times.

### Cell Cycle Analysis

Cell Cycle Analysis was performed by flow cytometry (FCM). Cells were collected and fixed with 70% ethanol at 4°C overnight, and then washed and stained with a mixed solution of propidium iodide and RNase A (9:1) (KGA511; KeyGen Biotech, Nanjing, China) for 1 h at room temperature. Immediately thereafter, the cell cycle was detected by CytoFLEX (Beckman Coulter, San Diego, CA, USA), and the results were analyzed with the CytExpert software.

### Quantitative Real-Time PCR (qRT-PCR)

Total RNA was extracted using TRIzol (15596018, Invitrogen), and cDNAs were synthesized using the SuperScript™ First-Strand Synthesis system (18091200, Invitrogen). qRT-PCR assays were performed using the Fast SYBR Green Master Mix kit (4385614, Applied Biosystems) in the Q6 real-time PCR system (Thermo Fisher Scientific, Waltham, MA, USA). The primers used in this assay were as follows: human CCDC68 (forward, 5′-TCTGCCTTGTATGAGTCTACGTCC-3′; reverse, 5′- A GGATCCATTTCAGAATCAGAGCC-3′), human CDK4 (forward, 5′-CTACAGCTACCAGATGGCACTTAC-3′; reverse, 5′-CAAAGATACAGCCAACACTCCACA-3′), human ITCH (forward, 5′-GGAAGCAACCCCTTACAGTTATC-3′; reverse, 5′-CTAATGCAGCAGTTCCCAACAA-3′), and human GAPDH (forward, 5′-GACTCATGACCACAGTCCATGC-3′; reverse, 5′-CAGGTCAGGTCCACCACTGA-3′).

### Dual-Luciferase Reporter Assay

HCT116 (Vector), HCT116 (CCDC68), RKO (Vector), and RKO (CCDC68) cells were transiently co-transfected with the ITCH promoter-driven luciferase reporter and pRL-TK for 24 h. After washing twice with PBS, cells were lysed with passive lysis buffer for 10 min at room temperature. Then, 20 μL cell lysate was transferred to a 96-well plate, and 40 μL Luciferase Assay Reagent II was added to detect ITCH promoter activity. Thereafter, Stop & Glo Buffer (Promega, USA) was added to each well to detect TK activity, which was measured in a Centro LB 960 luminometer (Berthold).

### Protein Degradation Assay

To evaluate the potential effects of CCDC68 on the degradation of CDK4 in CRC cells, 5 × 10^5^ stably transfected HCT116 (Vector) and HCT116 (CCDC68) cells were added into each well of a 6-well plate and cultured in complete medium at 37°C in a 5% CO_2_ incubator. The medium was replaced by 0.1% FBS medium after the cell density reached 50–60%, and cells were then starved for 12 h, followed by culture in 10% FBS medium for 12 h. Thereafter, cells were treated with medium containing 10 µM MG132 for 5 h before exposure to CHX (50 μg/mL) for 0, 3, 6, and 12 h. The protein stability of CDK4 was evaluated by western blotting at each time point.

### Immunoprecipitation

293T cells were transiently transfected with GFP-CDK4 plasmid. At 36 h after transfection, cells were harvested, and total cell lysates were extracted using cell lysis buffer (9803, Cell Signaling Technology, USA) containing a complete protein inhibitor cocktail (04693116001, Roche, Germany) on ice. The obtained lysates were incubated with rotation at 4°C for 30 min. After centrifugation, the supernatant (1000 μL) was divided into two aliquots: 450 μL (50 μL was removed, denatured, and stored to check the input by western blotting) was incubated with 2 µg anti-CDK4 antibody (sc-260); and 450 μL (50 μL was removed, denatured, and stored to check the input by western blotting) was incubated with a nonspecific antibody (rabbit IgG, sc-2749). After incubation overnight at 4°C, 30 µL protein A/G-coated magnetic beads (Thermo Fisher Scientific) were added to each sample and incubated for 2–4 h at 4°C. Extracts were sequentially washed five times with 1× Cell Lysis Buffer using a magnetic stand. Finally, 60 µL protein elution buffer and 0.6 µL DTT were added, and samples were heated at 37°C for 2 h to denature and separate the magnetic beads. The samples without magnetic beads were subjected to western blotting analysis.

### Xenograft Model in Nude Mice *In Vivo*

Animal experiments were performed in the animal institute of Wenzhou Medical University according to the protocols approved by the Laboratory Animal Center of Wenzhou Medical University and the Laboratory Animal Ethics Committee of Wenzhou Medical University, as described in our previous publication (21). Female BALB/c athymic nude mice (3 or 4 weeks old) were purchased from Shanghai Silaike Experimental Animal Company (license no. SCXK, Shanghai 2010-0002). After 1–2 weeks of acclimatization, mice were randomly allocated to one of two groups (five mice/group) and then subcutaneously injected into the right flank with 5 × 10^6^ HCT116 (Vector) or HCT116 (CCDC68) cells suspended in 100 μL Serum-free medium McCoy’s 5A. After 4 weeks, mice were sacrificed, and tumors were surgically removed, imaged, and weighed. Two third of the tumor was fixed in 4% paraformaldehyde for IHC, and the remaining third was frozen at -80°C to extract RNA if necessary.

### Bioinformatic Analysis

UbiBrowser database was used to screen possible E3 ligases related to CDK4 (http://ubibrowser.ncpsb.org/). The potential transcription factors binding to the ITCH promoter were predicted by the JASPAR database (http://jaspar.genereg.net/), with a profile score threshold of 95%.

### Statistical Analysis

All experimental data are expressed as the mean ± standard deviation (mean ± SD). Graphs and statistical analyses were performed using GraphPad Prism 6 (GraphPad Software, San Diego, CA, USA). The Kaplan–Meier method was used to draw the survival curve, and the log-rank test was used to compare the differences between groups. Comparisons between the control group and the experimental group were performed using the Student’s *t*-test. *P* < 0.05 was considered statistically significant.

## Results

### CCDC68 is Downregulated in CRC and Related to Patient Prognosis

**Figure 1:**
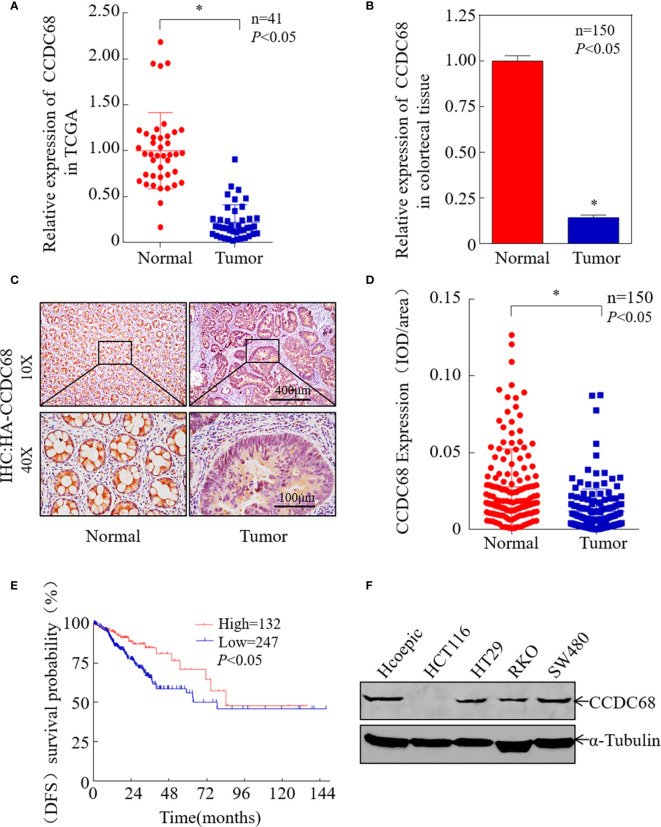
CCDC68 is downregulated in CRC tissues and cells. **(A)** CCDC68 mRNA levels in CRC according to the Cancer Genome Atlas (TCGA) data. **(B)** The mRNA expression level of CCDC68 in 150 pairs of CRC tissues and paracancerous tissues (normal). **(C)** Representative IHC images of CCDC68 protein expression in CRC tissues and adjacent normal tissues. **(D)** Quantification of CCDC68 expression in 150 pairs of CRC tissues and adjacent normal tissues. **(E)** CCDC68 mRNA expression levels and disease-free survival of patients from TCGA CRC data. **(F)** Expression of CCDC68 in human CRC cell lines (HCT116, HT29, RKO, and SW480) and in the normal colorectal epithelial cell line HCoEpiC. Data are presented as the mean ± SD, *P < 0.05.

To investigate the potential role of CCDC68 in human CRC progression, the Cancer Genome Atlas (TCGA) database was used to analyze the expression levels of CCDC68 in 41 pairs of cancerous and normal tissues from CRC patients. The results showed that CCDC68 was markedly downregulated in CRC (**Figure 1A**). Furthermore, we analyzed the expression levels of CCDC68 in 150 fresh CRC samples and matched adjacent (normal) colorectal tissues by qRT-PCR and IHC assays. The results showed that CCDC68 was significantly downregulated in tumor tissues, which was consistent with TCGA data (**Figures 1B–D**).

To assess the role of CCDC68 in CRC, we analyzed the relationship between CCDC68 expression levels and disease-free survival (DFS) data from the TCGA database. Kaplan–Meier survival analysis revealed that CCDC68 downregulation was associated with a poor prognosis in CRC patients (**Figure 1E**). In addition, CCDC68 expression was lower in human CRC cell lines (HCT116, HT29, RKO, and SW480) than that in the human immortalized normal colorectal epithelial cell line HCoEpiC (**Figure 1F**).

### CCDC68 Overexpression Suppresses Human CRC Cell Growth *In Vitro* and *In Vivo*

**Figure 2:**
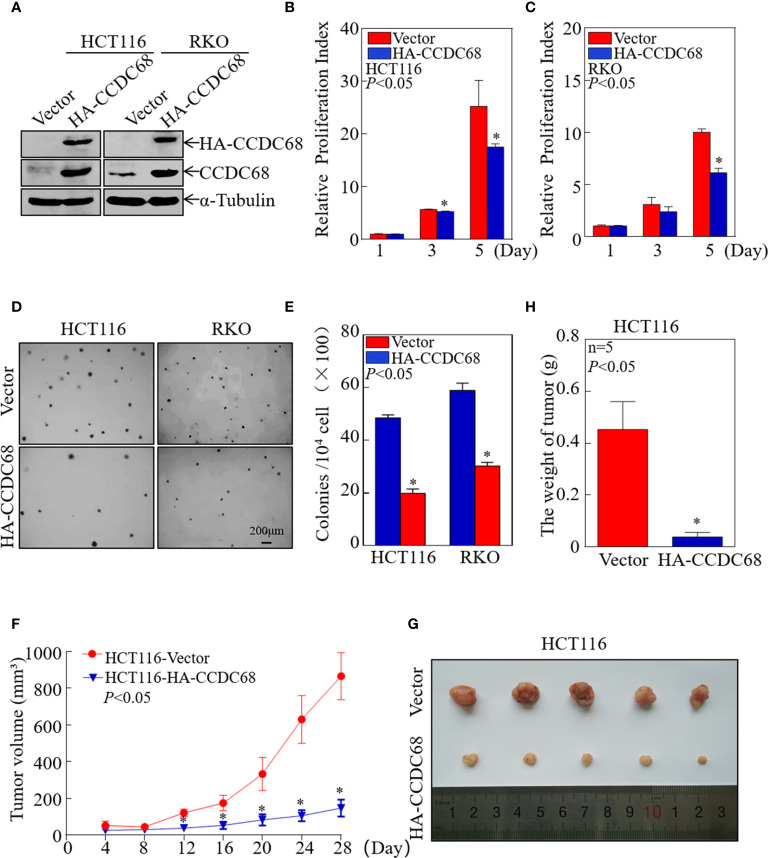
CCDC68 overexpression inhibits CRC cell growth in vitro and in vivo. **(A)** Stable transfectants of HCT116 (Vector) vs. HCT116 (HA-CCDC68) and RKO (Vector) vs. RKO (HA-CCDC68) were identified by western blotting. **(B, C)** Cell proliferation was detected by ATP assay. Data were analyzed Student′s *t*-test. **(D, E)** Anchorage-independent growth was examined by soft agar assay. Cell colonies were counted, and data were presented as colonies per 10,000 cells and analyzed by Student′s *t*-test. **(F)** Tumor growth curve for each group. Data were analyzed by Student′s *t*-test. **(G)** Representative images of tumors in each group. **(H)** Statistical analysis of tumor mass in each group; n = 5, Student′s *t*-test. Data are presented as the mean ± SD. *P < 0.05.

To evaluate whether CCDC68 is involved in the development of CRC, we constructed four stable transfectants, HCT116 (CCDC68), HCT116 (Vector), RKO (CCDC68), and RKO (Vector), which were examined by western blotting (**Figure 2A**). CCDC68 overexpression significantly decreased the monolayer growth of HCT116 and RKO cells compared with that of control vector transfectants (**Figures 2B, C**). CCDC68 overexpression also significantly decreased the anchorage-independent growth of CRC cells (**Figures 2D, E**). These data indicate that CCDC68 inhibited CRC cell growth *in vitro*.

To extend our findings *in vivo*, a xenograft nude mouse model was established by subcutaneous injection of equal numbers of HCT116 (Vector) and HCT116 (CCDC68) cells into nude mice. Tumor size was measured periodically, and a tumor growth curve was drawn (**Figure 2F**). The results showed that the weight and size of the subcutaneous tumors were significantly reduced in mice injected with HCT116 (CCDC68) cells than that in the HCT116 (Vector) control group (**Figures 2G, H**). These data confirmed that overexpression of CCDC68 decreased the growth ability of CRC cells.

### Overexpression of CCDC68 Induces G0/G1 Phase Arrest in CRC Cells and Downregulates the Expression of the CDK4 Protein

**Figure 3:**
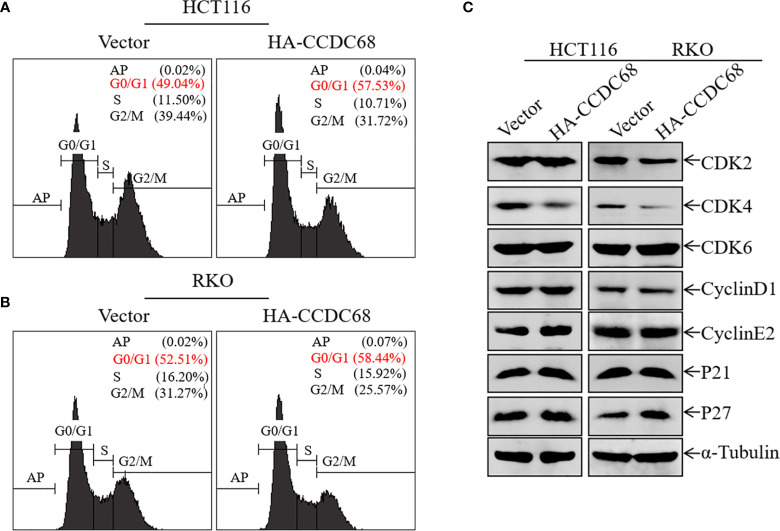
Overexpression of CCDC68 induces G0/G1 phase arrest and downregulates CDK4 in CRC cells. **(A, B)** Cell cycle of HCT116 (Vector/HA-CCDC68) and RKO (Vector/HA-CCDC68) cells examined by flow cytometry. **(C)** Expression of positive cell cycle regulators in the G0/G1 phase of HCT116 (Vector/HA-CCDC68) and RKO (Vector/HA-CCDC68) cells.

Uncontrolled cell cycle progression and cell proliferation are two important biological characteristics of tumor cells (22). We therefore used FCM to analyze cell cycle progression in HCT116 (Vector or CCDC68) and RKO (Vector or CCDC68) cells, and to explore the potential molecular mechanism underlying the effect of CCDC68 on inhibiting CRC cell growth. As shown in **Figures 3A, B**, CCDC68 overexpression induced cell cycle G0/G1 arrest, suggesting that the inhibition of CRC cell growth by CCDC68 is related to its ability to induce G0-G1 growth arrest.

To elucidate the molecular mechanism underlying the effect of CCDC68 on inducing G0/G1growth arrest in CRC cells, the expression of G0/G1 phase-related proteins was detected in HCT116 and RKO cells by western blotting. Ectopic expression of CCDC68 did not significantly affect the expression of the cell cycle regulatory proteins CDK2, CDK6, cyclin D1, cyclin E2, P21, and P27 (**Figure 3C**). However, CDK4 expression was lower in HCT116 (CCDC68) and RKO (CCDC68) cells than in the corresponding control cells. These results suggest that CDK4 is a key downstream molecule associated with the inhibition of CRC cell proliferation by CCDC68.

### CCDC68 Inhibits the Proliferation of CRC Cells by Targeting CDK4

**Figure 4:**
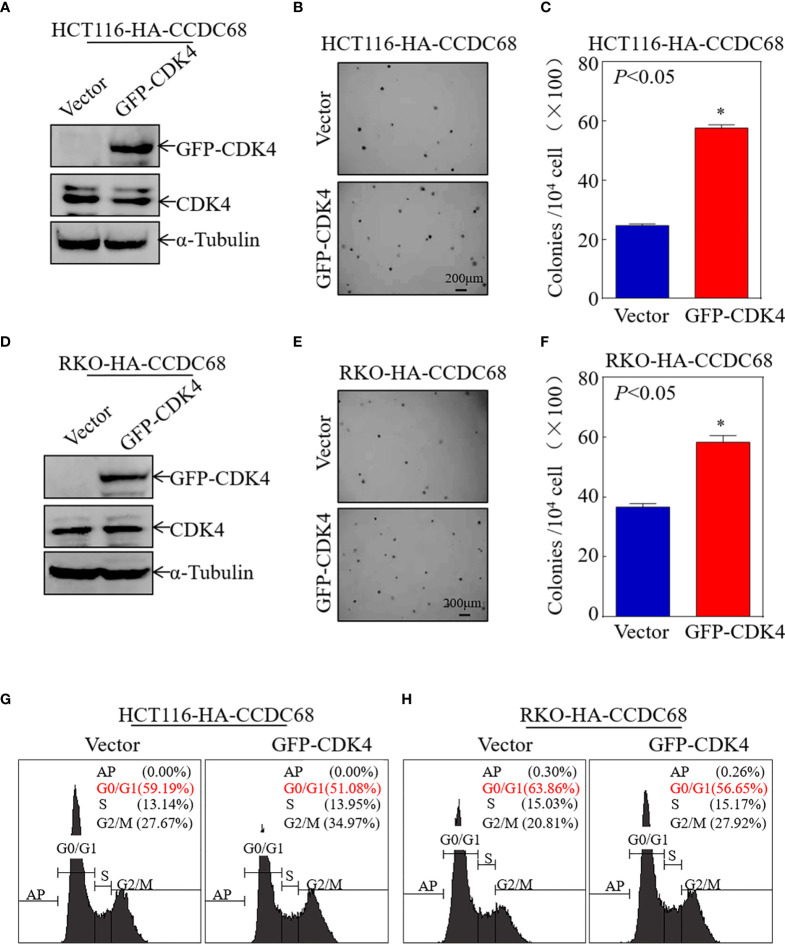
CCDC68 inhibits malignant CRC cell proliferation by downregulating CDK4. **(A, D)** Stable transformation efficacy was assessed by western blotting in HCT116 (CCDC68) and RKO (CCDC68) cells overexpressing GFP-CDK4. **(B, E)** A soft agar colony formation assay was used to detect the effect of CDK4 on the proliferation of CRC cells. **(C, F)** Diagrams comparing the number of cell clones between the control and experimental groups. **(G, H)** Effect of CDK4 on the G0/G1 phase in HCT116 (CCDC68) and RKO (CCDC68) cells. Data are presented as the mean ± SD and analyzed by Student′s *t*-test, *P < 0.05.

To further explore the role of CDK4 as a downstream effector of CCDC68, CDK4 was ectopically expressed in HCT116 (CCDC68) and RKO (CCDC68) cells (**Figures 4A, D**). The effect of CDK4 overexpression on the anchorage-independent proliferation of HCT116 (CCDC68) and RKO (CCDC68) cells was examined by soft agar assay. As shown in **Figures 4B, C, E, F**, ectopic expression of GFP-CDK4 increased anchorage-independent growth. The results of FCM analysis confirmed that CCDC68-induced G0/G1 growth arrest was reversed by CDK4 overexpression compared with that in the control vector (**Figures 4G, H**). Collectively, these results suggest that CDK4 is a downstream regulator mediating the inhibition of CRC cell proliferation by CCDC68.

### ITCH might Serve as an E3 Ubiquitin Ligase Targeting CDK4 for Degradation

**Figure 5:**
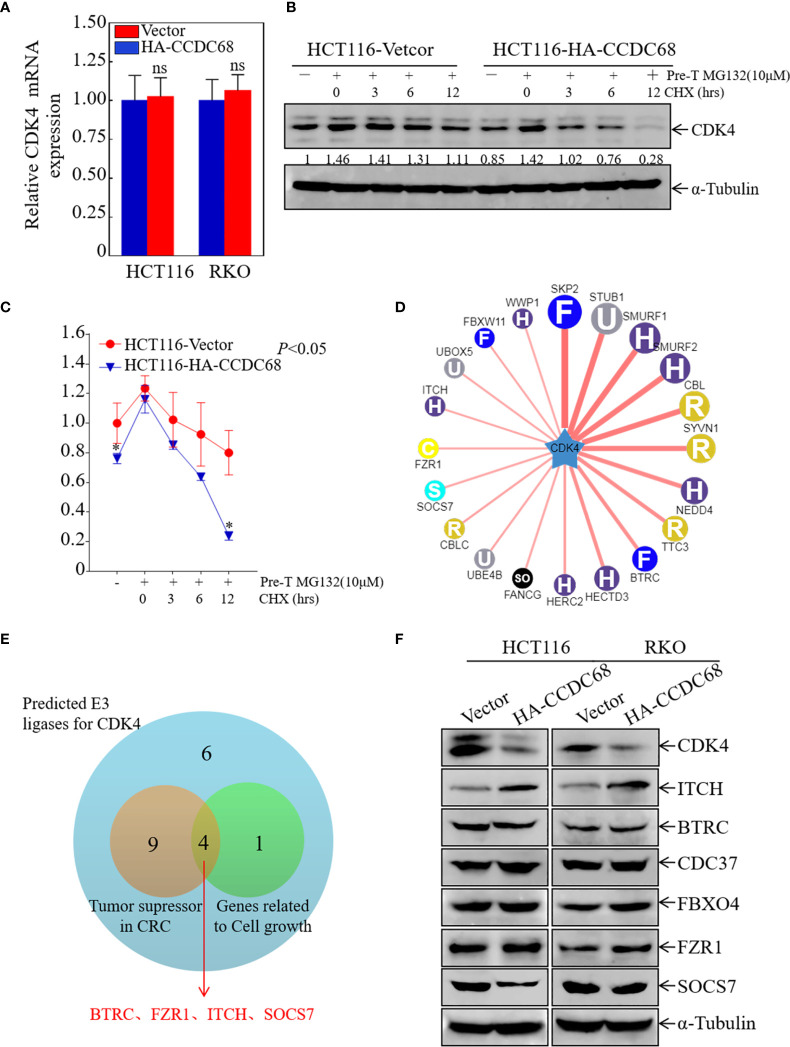
ITCH may function as an E3 ubiquitin ligase targeting CDK4 for degradation. **(A)** Relative CDK4 mRNA expression in HCT116 (Vector/HA-CCDC68) and RKO (Vector/HA-CCDC68) cells. Student′s *t*-test, ns, not significant. **(B)** CDK4 protein degradation was monitored in HCT116 (CCDC68) and HCT116 (Vector) cells pretreated with MG132 for 5 h followed by CHX for various times. **(C)** CDK4 protein degradation rates in HCT116 (CCDC68) and HCT116 (Vector) cells. Three independent degradation experiments were performed, and the results were analyzed using ImageJ software and analyzed by Student′s *t*-test. *P < 0.05. **(D)** Molecules potentially involved in the regulation of CDK4 protein degradation were predicted by the UbiBrowser database. **(E)** Venn diagram screening the E3 enzymes involved in the regulation of CDK4 protein degradation. **(F)** Expression of CDK4, ITCH, BTRC, CDC37, FBXO4, and SOCS7 in HCT116 (Vector/HA-CCDC68) and RKO (Vector/HA-CCDC68) cells.

To elucidate the molecular mechanism underlying the effect of CCDC68 on downregulating CDK4 expression, the mRNA levels of CDK4 were measured in HCT116 (CCDC68) and RKO (CCDC68) cells by qRT-PCR. The results showed that the mRNA levels of CDK4 did not differ significantly between the CCDC68 overexpression group and the control group **(** **Figure 5A**). This suggests that CCDC68 regulates CDK4 expression at the protein level rather than at the mRNA level. Cells were treated with MG132 (10 µM) and CHX (50 μg/mL) (23), and harvested at different time points, and CDK4 protein levels were detected by western blotting. As shown in **Figures 5B, C**, the rate of degradation of the CDK4 protein was significantly higher in HCT116 cells overexpressing CCDC68 than in the control group. These results indicate that CCDC68 downregulates CDK4 by promoting its ubiquitination.

Next, we examined the mechanism by which CCDC68 regulates the ubiquitination of CDK4. FBXO4 is an E3 ubiquitin ligase for CDK4 (24), and cell division cycle 37 (Cdc37), a partner of heat stress protein 90 (HSP90), enhances CDK4 stability and promotes CRC cell survival (25). In addition, we used the UbiBrowser database to screen for possible E3 ligases related to CDK4 degradation (**Figure 5D**). Potential E3 enzymes acting as tumor suppressors in CRC and related to cell growth were selected from the top 20 E3 enzymes identified (**Figure 5E**), and the expression levels of these proteins were examined in HCT116 and RKO cells overexpressing CCDC68 by western blotting. The results showed that the expression of ITCH was significantly higher in the HCT116 (CCDC68) and RKO (CCDC68) transfectants than in the control groups, whereas other enzymes showed no significant differences in expression (**Figure 5F**). We speculated that CCDC68 may accelerate the degradation rate of CDK4 through ITCH, ultimately inhibiting the malignant proliferation of CRC cells.

### CCDC68 Promotes the Degradation of CDK4 by Upregulating ITCH Expression

**Figure 6:**
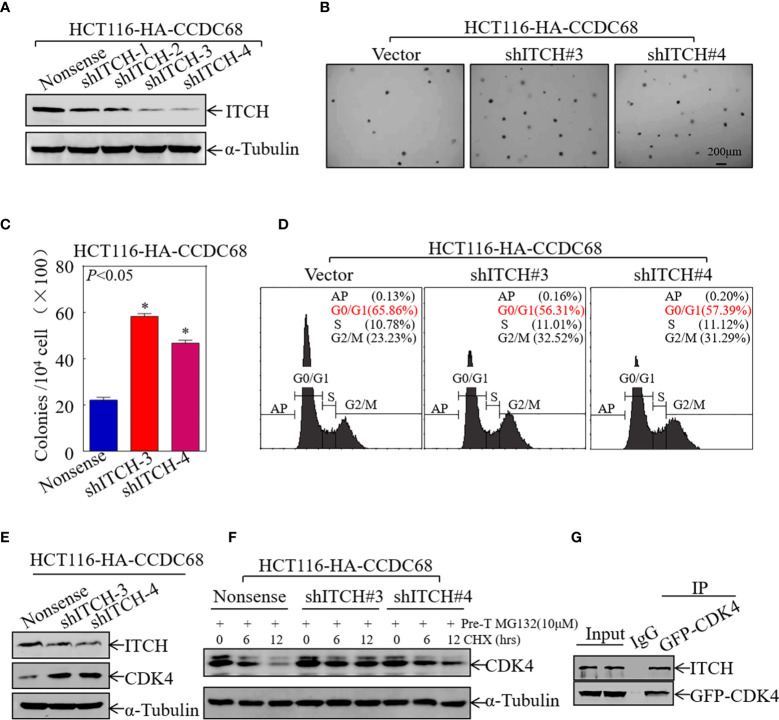
CCDC68 accelerates the degradation of CDK4 by regulating ITCH expression. **(A)** Western blotting analysis of the efficacy of HCT116 (CCDC68) transfection with shITCH lentivirus or Nonsense lentivirus. **(B, C)** HCT116 (CCDC68/shITCH#3, #4) and HCT116 (CCDC68/Nonsense) cells were tested for anchorage-independent growth. Cell colonies were counted, and the results are presented in the diagram. Data are presented as the mean ± SD and analyzed by Student′s *t*-test, *P < 0.05. **(D)** Analysis of HCT116 (CCDC68/shITCH#3, #4) and HCT116 (CCDC68/Nonsense) cell cycles by flow cytometry. **(E)** Western blotting analysis of CDK4 levels in HCT116 (CCDC68/shITCH #3 and #4), and HCT116 (CCDC68/Nonsense) cells. **(F)** Effect of ITCH knockdown on CDK4 degradation rate in HCT116 (CCDC68) cells detected by western blotting. **(G)** The interaction between CDK4 and ITCH was analyzed by co-immunoprecipitation in 293T cells transfected with the GFP-CDK4 plasmid.

To verify the role of ITCH in CCDC68-regulated cell growth, four stable ITCH knockdown clones of HCT116 (CCDC68) cells were established (**Figure 6A**). The effect of ITCH knockdown on cell proliferation and cell cycle progression was examined by soft agar assay and FCM in HCT116 (CCDC68) cells. The results showed that ITCH knockdown markedly reversed the inhibitory effects of CCDC68 on HCT116 anchorage-independent growth and G0/G1cell cycle arrest (**Figures 6B–D**).

To further investigate whether ITCH mediates CDK4 degradation, the effect of ITCH knockdown on CDK4 expression was examined by western blotting in HCT116 (CCDC68) cells; a protein degradation assay and IP assay were also performed. As shown in **Figures 6E, F**, knockdown of ITCH in HCT116 (CCDC68) cells significantly upregulated CDK4 and dramatically decreased the rate of CDK4 protein decay. The results of IP with an antibody that specifically pulls down the CDK4 protein showed that ITCH was present in the immune complex (**Figure 6G**), suggesting that ITCH binds to and interacts with CDK4 to trigger its degradation. These results suggest that CCDC68 promotes CDK4 protein degradation by regulating ITCH protein expression, thereby inhibiting the malignant proliferation of CRC cells.

### CCDC68 Promotes ITCH Transcription by Upregulating RXRα Protein Expression

**Figure 7:**
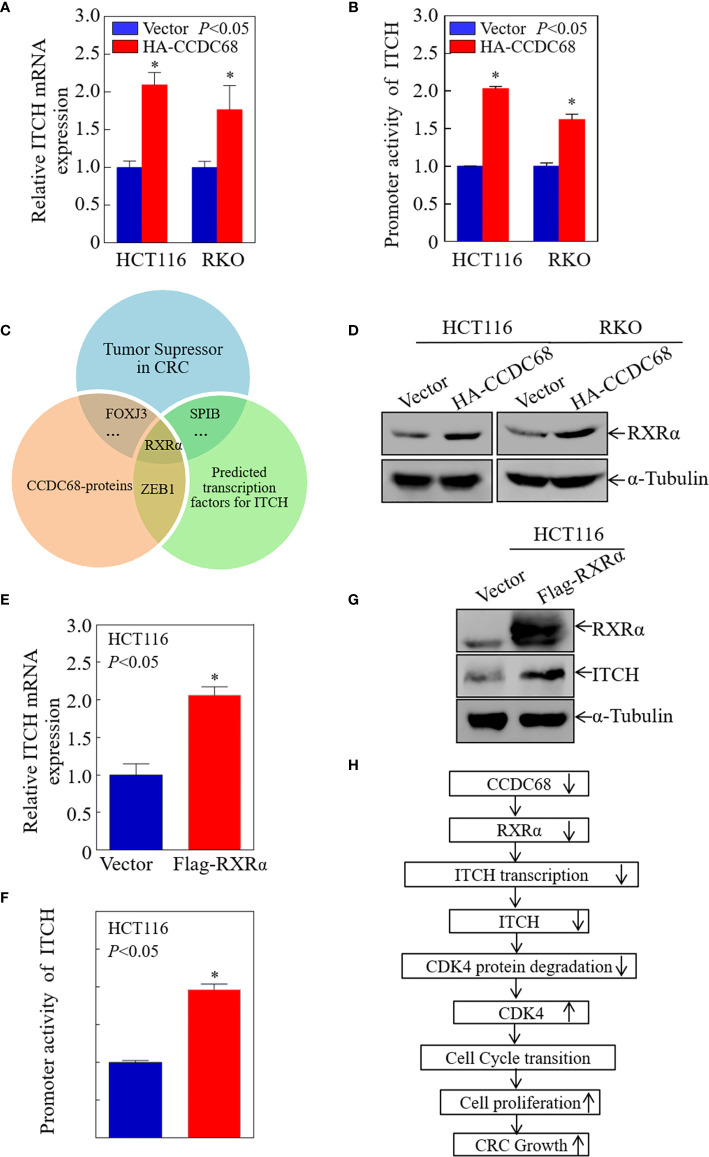
CCDC68 promotes ITCH transcription by upregulating RXRα expression. **(A)** CCDC68 overexpression increased the mRNA levels of ITCH. **(B)** ITCH promoter activity in CCDC68 overexpression cells compared with vector control cells. **(C)** Bioinformatics and proteomics analysis of transcription factors potentially involved in the regulation of ITCH transcription. **(D)** RXRα expression levels in HCT116 (Vector/HA-CCDC68) and RKO (Vector/HA-CCDC68) cells. **(E)** ITCH mRNA levels in HCT116(RXRα) and HCT116(Vector) cells. **(F)** ITCH promoter activity in HCT116 (RXRα) and HCT116 (Vector) cells. **(G)** Expression levels of ITCH in HCT116 (RXRα) and HCT116 (Vector) cells. **(H)** Schematic diagram of the molecular mechanism underlying the effect of CCDC68 on regulating the proliferation of colorectal cancer cells. Data are presented as the mean ± SD and analyzed by Student′s *t*-test. *P < 0.05.

To elucidate the potential mechanisms underlying the upregulation of ITCH by CCDC68 in CRC cells, the mRNA expression level of ITCH was measured in transfectants. As shown in **Figure 7A**, ITCH mRNA levels were markedly higher in CCDC68-overexpressing stable transfectants than in vector transfectants, suggesting that the upregulation of ITCH occurs at the transcriptional level or by modulating mRNA stability.

To determine whether CCDC68 regulates ITCH promoter activity, we detected the promoter activity of ITCH in HCT116 (CCDC68) and RKO (CCDC68) cells, and in the corresponding control groups using a dual-luciferase reporter assay. The results showed that CCDC68 overexpression significantly promoted the transcriptional activity of the ITCH promoter (**Figure 7B**), indicating that CCDC68 upregulates ITCH expression at the transcriptional level. Transcription factors regulate the expression of target genes by binding to the promoter region. Therefore, we predicted the transcription factors that may bind to the ITCH promoter using the JASPAR dataset with a profile score threshold of 95%.

Next, quantitative proteomics analysis was performed to identify the transcription factor involved in the regulation of ITCH expression by CCDC68. The transcription factors that play an inhibitory role in CRC were identified, and the data were combined with the results of website prediction and proteomics analysis. The results indicated that RXRα might be involved in the regulation of ITCH expression by CCDC68 at the transcriptional level (**Figure 7C**). Analysis of RXRα expression in HCT116 (CCDC68) and RKO (CCDC68) cells and control cells by western blotting showed that RXRα was upregulated in CCDC68-overexpressing cells (**Figure 7D**). RXRα was overexpressed in HCT116 cells, and the mRNA level, promoter activity, and protein levels of ITCH were analyzed. RXRα overexpression significantly increased ITCH mRNA levels, promoter activity, and the expression of the ITCH protein (**Figures 7E–G**). Collectively, these results identified RXRα as a critical transcription factor involved in the regulation of ITCH transcription by CCDC68.

## Discussion

CCDC68 is upregulated in NSCLC, and downregulating CCDC68 expression decreases cell proliferation and increases apoptosis, suggesting that CCDC68 is a candidate biomarker for the detection of malignant transformation in lung cancer (16). However, CCDC68 plays a tumor-inhibitory role in pancreatic cancer (15), which is inconsistent with the tumor-promoting function of CCDC68 in NSCLC. This suggests that CCDC68 plays different roles in various human cancers. CCDC68 copy number is decreased in CRC (17), although its specific biological function and underlying molecular mechanism remain unclear. In this study, we confirmed the role of CCDC68 in inhibiting the growth of human CRC cells, and clarified the molecular mechanism underlying the effect of CCDC68 on the progression of CRC. The results showed that CCDC68 expression was lower in human CRC than in paired non-tumor tissues, and analyses of a human CRC database and cell lines supported the low expression of CCDC68 in CRC. The data suggested that CCDC68 acts as a tumor suppressor in CRC and has a potential prognostic role in predicting survival.

Overexpression of CCDC68 inhibited the monolayer growth and adhesion-independent growth of CRC cells *in vitro*, and the growth of transplanted tumors *in vivo*. In addition, CCDC68 promoted the transcription of ITCH by upregulating the transcription factor RXRα, and increased levels of ITCH downregulated CDK4 expression by inducing its degradation. Thus, CCDC68 inhibits the malignant transformation of cells by inhibiting G0/G1 cell cycle phase transition, thereby playing an important role in inhibiting the growth of CRC. These findings provide important insight into the role of CCDC68 in the development of human CRC. Consistent with the inhibitory function of CCDC68 in pancreatic cancer, it plays a tumor suppressor role in CRC, providing a potential new target for early diagnosis and treatment of CRC.

CDKs are endogenous cell cycle regulators that modulate cell division and proliferation (26). CDK4 is an important regulator of the G1 phase of the cell cycle. CDK4/6 bind to cyclin D to form a kinase-active complex, which phosphorylates retinoblastoma protein and drives the progression from G1 to S phase (27). Several studies have demonstrated that CDK4 is upregulated in cancer (28–33) and inhibiting CDK4 expression increases the efficacy of clinical treatments for breast cancer, melanoma, liposarcoma, and mantle cell lymphoma (34–37). This indicates that CDK4 could be a target for the treatment of malignant tumors. However, current CDK4 inhibitors are not clinically effective in treating CRC. Therefore, looking for specific inhibitors that regulate CDK4, developing highly selective drugs for CDK4, and preparing reasonable and effective combined strategies for specific patients are of great significance for improving the clinical treatment effect of CRC patients (38). However, the mechanism underlying the regulation of CDK4 protein degradation in human CRC has not been fully elucidated. Ubiquitin-mediated degradation is essential for controlling CDKs. Studies have shown that PAQR4 controls the steady-state level of CDK4 by regulating the Skp2-mediated ubiquitination of CDK4 (24). In CRC, Cdc37 activates the RB1 signaling pathway by increasing the stability of CDK4, which plays a key role in promoting the survival of CRC cells (25). In addition, Bury et al. showed that DUX4, a direct inhibitor of CDK1 activity, can also bind to CDK4, but it is unclear whether DUX4 inhibits CDK4 activity (39). The present results indicate that CCDC68 is a key upstream regulator of CDK4 in CRC. CCDC68 overexpression accelerated the degradation rate of the CDK4 protein. A bioinformatics screening of E3 ubiquitin ligases interacting with CDK4 showed that ITCH was involved in CDK4 degradation mediated by CCDC68.

Ubiquitination is a post-translational modification involved in the regulation of signaling pathways (40), and E3 ubiquitin ligases catalyze the ubiquitination of target proteins (41). ITCH is a member of the Nedd4 family of HECT-type E3 ligases, which mediate the ubiquitination of multiple targets (42–44). ITCH acts as a cancer-promoting factor in breast cancer, pancreatic cancer, hepatocellular carcinoma, and chronic lymphocytic leukemia (45–47). However, studies analyzing the expression and function of ITCH in CRC show that ITCH is downregulated in CRC and acts as a tumor suppressor by inhibiting the Wnt/β-catenin pathway (48, 49). In addition, Kathania et al. found that ITCH can inhibit IL-17-mediated colon inflammation and tumorigenesis through ROR-γt ubiquitination (50), and Ko et al. reported that ITCH can form a destruction complex to antagonize tumor necrosis factor receptor I (TNFRI), thereby inhibiting TNF-NF-κB signal transduction and tumorigenesis (51). In this study, we found that overexpression of CCDC68 significantly upregulated ITCH and downregulated CDK4 in CRC cells, resulting in G0/G1 phase arrest. Knockdown of ITCH in HCT116 (CCDC68) cells decreased the degradation rate of the CDK4 protein, thereby promoting the formation and proliferation of CRC cell colonies. Co-immunoprecipitation assays confirmed the interaction between ITCH and CDK4, revealing a new mechanism underlying the role of CCDC68 in regulating CDK4 protein degradation through ITCH. The results indicated that ITCH acts as a downstream effector of CCDC68 to inhibit the growth of CRC, which is consistent with reports that ITCH plays a negative regulatory role in the progression of CRC (48, 49). Taken together, these findings indicate that upregulating ITCH may provide therapeutic benefits for CRC patients.

The regulation of ITCH expression has not been studied extensively. Studies show that ITCH expression is regulated by microRNAs in cancer (46–52). We showed that CCDC68 upregulated the expression of ITCH at the transcriptional level. RXRα is a member of the nuclear receptor (NR) superfamily, which functions in the regulation of transcription, and in controlling the development, homeostasis, and metabolism of organisms (53). RXRα is downregulated in cervical cancer and inhibits its progression (54). In this study, proteomics analysis and bioinformatics prediction showed that the ITCH promoter contains a DNA binding site for RXRα, and ITCH and RXRα were consistently upregulated. Western blotting experiments indicated that CCDC68 overexpression significantly increased the levels of RXRα, suggesting that RXRα is involved in the transcription of ITCH. Low expression levels of RXRα are closely related to the pathogenesis and progression of CRC (55). Volate et al. reported that low concentrations of green tea are sufficient to de-silence RXRα and inhibit intestinal tumorigenesis in the Apc^Min/+^ mouse (56). Therefore, epigenetic regulation of RXRα may be a new strategy for the prevention and treatment of CRC. However, the exact mechanisms underlying the regulation of RXRα by CCDC68 need to be further studied.

In summary, as shown in **Figure 7H**, the present study identified a new CCDC68/RXRα/ITCH/CDK4 regulatory axis involved in CRC progression. CCDC68 was downregulated in CRC, and functional experiments showed that CCDC68 inhibited CRC cell growth *in vitro* and tumor formation *in vivo*. CCDC68 overexpression increased the transcription and expression of ITCH by upregulating RXRα, thereby promoting the binding of ITCH to CDK4 and the degradation of CDK4. This led to the inhibition of G0/G1 phase transition and cell growth in human CRC cells. The present results indicate that CCDC68 is an important tumor suppressor molecule in CRC, and CCDC68 and its downstream effectors may become potential targets for the early diagnosis and/or treatment of CRC.

## Data Availability Statement

The datasets presented in this study can be found in online repositories. The names of the repository/repositories and accession number(s) can be found below: http://www.ebi.ac.uk/pride/archive/projects/PXD024343.

## Ethics Statement

The studies involving human participants were reviewed and approved by Ethics Committee of Wenzhou Medical University. Written informed consent for participation was not required for this study in accordance with the national legislation and the institutional requirements. The animal study was reviewed and approved by Institutional Animal Care and Use Committee of Wenzhou Medical University.

## Author Contributions

CW, LZ and HH conceived and designed the study. CW, HL, ZJ, YZ, ZF and XJ detected the cells′ biological function, performed the RT-PCR assays, carried out the soft agar, ATP assay, Western blotting, and luciferase reporter assays, and conducted the statistical analyses. XZ and LW analyzed clinical samples. CW, LZ and HH drafted the manuscript. All authors contributed to the article and approved the submitted version.

## Funding

This work was partially supported by Key Project of Science and Technology Innovation Team of Zhejiang Province (2013TD10), Wenzhou Medical University (89216021), Wenzhou Science & Technology Bureau (Y20180857), Key Discipline of Zhejiang Province in Medical Technology (First Class, Category A), and Xinmiao Talent Program of Zhejiang Province (2020R413065).

## Conflict of Interest

The authors declare that the research was conducted in the absence of any commercial or financial relationships that could be construed as a potential conflict of interest.
