# Supplementary material for: Coiled-Coil Domain-Containing 68 Downregulation Promotes Colorectal Cancer Cell Growth by Inhibiting ITCH-Mediated CDK4 Degradation
Source: Front Oncol. 2021 Apr 22;11:668743. doi: 10.3389/fonc.2021.668743 (PMC8100586; doi:10.3389/fonc.2021.668743)
Supplement: Supplementary file 1 [file Table_1.docx]

Supplementary Material

**Supplementary Table1.** The information of Colorectal cancer patients including case number, gender, age and tumor type.

| case（NO#） | gender | age | stage | case（NO#） | gender | age | stage |
| --- | --- | --- | --- | --- | --- | --- | --- |
| 1# | female | 34 | Ⅳ | 35# | male | 76 | Ⅰ |
| 2# | female | 52 | Ⅲ | 36# | female | 66 | Ⅲ |
| 3# | female | 69 | Ⅰ | 37# | female | 52 | Ⅲ |
| 4# | male | 47 | Ⅳ | 38# | male | 67 | Ⅳ |
| 5# | male | 60 | Ⅱ | 39# | female | 68 | Ⅰ |
| 6# | female | 69 | Ⅰ | 40# | male | 58 | Ⅰ |
| 7# | male | 62 | Ⅲ | 41# | male | 65 | Ⅳ |
| 8# | male | 79 | Ⅰ | 42# | female | 65 | Ⅱ |
| 9# | male | 77 | Ⅳ | 43# | male | 64 | Ⅱ |
| 10# | male | 74 | Ⅰ | 44# | male | 73 | Ⅰ |
| 11# | female | 55 | Ⅰ | 45# | male | 70 | Ⅱ |
| 12# | male | 20 | Ⅰ | 46# | female | 69 | Ⅲ |
| 13# | male | 52 | Ⅱ | 47# | male | 74 | Ⅱ |
| 14# | female | 47 | Ⅲ | 48# | male | 71 | Ⅳ |
| 15# | female | 61 | Ⅱ | 49# | female | 67 | Ⅳ |
| 16# | male | 59 | Ⅳ | 50# | male | 60 | Ⅱ |
| 17# | male | 68 | Ⅳ | 51# | female | 70 | Ⅱ |
| 18# | male | 80 | Ⅰ | 52# | male | 65 | Ⅲ |
| 19# | male | 53 | Ⅰ | 53# | male | 66 | Ⅲ |
| 20# | male | 69 | Ⅲ | 54# | female | 67 | Ⅰ |
| 21# | male | 51 | Ⅳ | 55# | female | 50 | Ⅳ |
| 22# | male | 63 | Ⅳ | 56# | female | 67 | Ⅳ |
| 23# | female | 64 | Ⅱ | 57# | male | 71 | Ⅲ |
| 24# | female | 47 | Ⅰ | 58# | female | 31 | Ⅲ |
| 25# | female | 61 | Ⅰ | 59# | female | 77 | Ⅲ |
| 26# | female | 55 | Ⅰ | 60# | male | 61 | Ⅰ |
| 27# | female | 49 | Ⅲ | 61# | male | 69 | Ⅰ |
| 28# | male | 64 | Ⅱ | 62# | male | 72 | Ⅳ |
| 29# | male | 71 | Ⅰ | 63# | female | 58 | Ⅳ |
| 30# | female | 81 | Ⅲ | 64# | female | 64 | Ⅱ |
| 31# | female | 44 | Ⅳ | 65# | female | 63 | Ⅱ |
| 32# | male | 57 | Ⅰ | 66# | male | 59 | Ⅱ |
| 33# | male | 66 | Ⅰ | 67# | female | 81 | Ⅰ |
| 34# | female | 65 | Ⅲ | 68# | male | 46 | Ⅱ |
| 69# | female | 63 | Ⅱ | 110# | male | 71 | Ⅳ |
| 70# | male | 74 | Ⅱ | 111# | male | 87 | Ⅳ |
| 71# | male | 52 | Ⅲ | 112# | female | 80 | Ⅲ |
| 72# | female | 76 | Ⅲ | 113# | male | 64 | Ⅱ |
| 73# | male | 59 | Ⅰ | 114# | female | 74 | Ⅳ |
| 74# | male | 64 | Ⅱ | 115# | male | 51 | Ⅳ |
| 75# | male | 71 | Ⅲ | 116# | female | 65 | Ⅲ |
| 76# | male | 71 | Ⅲ | 117# | male | 82 | Ⅱ |
| 77# | female | 65 | Ⅲ | 118# | male | 76 | Ⅱ |
| 78# | female | 66 | Ⅲ | 119# | male | 75 | Ⅳ |
| 79# | female | 67 | Ⅱ | 120# | male | 64 | Ⅳ |
| 80# | male | 64 | Ⅱ | 121# | male | 68 | Ⅱ |
| 81# | male | 78 | Ⅱ | 122# | male | 62 | Ⅰ |
| 82# | male | 56 | Ⅱ | 123# | female | 80 | Ⅲ |
| 83# | male | 67 | Ⅲ | 124# | male | 59 | Ⅰ |
| 84# | male | 78 | Ⅰ | 125# | female | 71 | Ⅰ |
| 85# | male | 55 | Ⅲ | 126# | male | 36 | Ⅲ |
| 86# | male | 65 | Ⅲ | 127# | male | 59 | Ⅲ |
| 87# | male | 54 | Ⅲ | 128# | female | 42 | Ⅲ |
| 88# | male | 42 | Ⅲ | 129# | female | 84 | Ⅲ |
| 89# | female | 47 | Ⅰ | 130# | male | 62 | Ⅳ |
| 90# | male | 75 | Ⅱ | 131# | male | 70 | Ⅲ |
| 91# | male | 79 | Ⅰ | 132# | male | 79 | Ⅱ |
| 92# | male | 70 | Ⅰ | 133# | male | 61 | Ⅲ |
| 93# | male | 53 | Ⅳ | 134# | male | 60 | Ⅰ |
| 94# | male | 46 | Ⅰ | 135# | female | 57 | Ⅱ |
| 95# | male | 73 | Ⅲ | 136# | male | 65 | Ⅱ |
| 96# | male | 70 | Ⅲ | 137# | male | 73 | Ⅲ |
| 97# | female | 49 | Ⅰ | 138# | male | 77 | Ⅱ |
| 98# | male | 73 | Ⅰ | 139# | female | 80 | Ⅲ |
| 99# | female | 77 | Ⅱ | 140# | female | 69 | Ⅰ |
| 100# | female | 68 | Ⅰ | 141# | female | 70 | Ⅳ |
| 101# | male | 43 | Ⅱ | 142# | male | 65 | Ⅳ |
| 102# | female | 76 | Ⅱ | 143# | male | 68 | Ⅱ |
| 103# | male | 71 | Ⅳ | 144# | male | 84 | Ⅱ |
| 104# | male | 75 | Ⅲ | 145# | male | 57 | Ⅰ |
| 105# | female | 79 | Ⅱ | 146# | female | 49 | Ⅱ |
| 106# | female | 52 | Ⅱ | 147# | female | 65 | Ⅲ |
| 107# | male | 40 | Ⅱ | 148# | female | 77 | Ⅰ |
| 108# | male | 69 | Ⅰ | 149# | female | 73 | Ⅲ |
| 109# | male | 47 | Ⅱ | 150# | female | 74 | Ⅲ |
